# ILF2 and ILF3 are autoantigens in canine systemic autoimmune disease

**DOI:** 10.1038/s41598-018-23034-w

**Published:** 2018-03-19

**Authors:** Hanna D. Bremer, Nils Landegren, Ronald Sjöberg, Åsa Hallgren, Stefanie Renneker, Erik Lattwein, Dag Leonard, Maija-Leena Eloranta, Lars Rönnblom, Gunnel Nordmark, Peter Nilsson, Göran Andersson, Inger Lilliehöök, Kerstin Lindblad-Toh, Olle Kämpe, Helene Hansson-Hamlin

**Affiliations:** 1 Department of Clinical Sciences, Swedish University of Agricultural Sciences, SE-750 07 Uppsala, Sweden; 2 Department of Medicine Solna, CMM, L8:01, Karolinska University Hospital, Karolinska Institutet, SE-171 76 Stockholm, Sweden; 3 Science for Life Laboratory, Department of Medical Sciences, Uppsala University, Uppsala, Sweden; 4 Affinity Proteomics, SciLifeLab, School of Biotechnology, KTH Royal Institute of Technology, SE-171 21 Solna, Sweden; 5 Euroimmun AG, 23560 Lübeck, Germany; 6 Departement of Medical Sciences, Rheumatology and Science for Life Laboratory, Uppsala University, SE 751 85 Uppsala, Sweden; 7 Department of Animal Breeding and Genetics, Swedish University of Agricultural Sciences, SE-750 07 Uppsala, Sweden; 8 Broad Institute of Harvard and MIT, Cambridge, MA 02142 USA; 9 Science for Life Laboratory, IMBIM, Uppsala University, SE-751 23 Uppsala, Sweden; 10 Department of Clinical Science and K.G. Jebsen Center for Autoimmune disorders, University of Bergen, 5021 Bergen, Norway; 11 Department of Medicine, Haukeland University Hospital, 5021 Bergen, Norway

## Abstract

Dogs can spontaneously develop complex systemic autoimmune disorders, with similarities to human autoimmune disease. Autoantibodies directed at self-antigens are a key feature of these autoimmune diseases. Here we report the identification of interleukin enhancer-binding factors 2 and 3 (ILF2 and ILF3) as autoantigens in canine immune-mediated rheumatic disease. The ILF2 autoantibodies were discovered in a small, selected canine cohort through the use of human protein arrays; a method not previously described in dogs. Subsequently, ILF3 autoantibodies were also identified in the same cohort. The results were validated with an independent method in a larger cohort of dogs. ILF2 and ILF3 autoantibodies were found exclusively, and at a high frequency, in dogs that showed a speckled pattern of antinuclear antibodies on immunofluorescence. ILF2 and ILF3 autoantibodies were also found at low frequency in human patients with SLE and Sjögren’s syndrome. These autoantibodies have the potential to be used as diagnostic biomarkers for canine, and possibly also human, autoimmune disease.

## Introduction

Autoimmune disorders are a diverse group of diseases affecting humans, dogs and other animals^1^. One important characteristic of autoimmune disease is the presence of autoantibodies directed against self-antigens. Autoantibodies can be pathogenic, for example, by forming immune-complexes that can deposit in different tissues and cause inflammation^2^. However they are not always direct pathogenic, but can in some instances be markers of cell-mediated autoimmunity^3^. In addition to providing insight into disease pathogenesis, autoantibodies can serve as diagnostic and prognostic markers^4–6^. Autoantibody analyses can also be used to stratify patients into etiologically homogenous subgroups, which can facilitate research and clinical management.

Dogs of several breeds are predisposed to develop complex autoimmune disorders, such as systemic lupus erythematosus (SLE) and other systemic rheumatic diseases^7–9^ and are considered a suitable model for studying complex diseases affecting both humans and dogs^10^. Although the term SLE is widely used in veterinary literature to describe a multi-systemic autoimmune disease, different diagnostic critera are used^11,12^, and the diseases are not necessarly identical, in humans and dogs. The Nova Scotia duck tolling retriever (NSDTR), is a breed particularly affected by immune-mediated rheumatic disease (IMRD)^13^. This is an SLE-related disease characterised by chronic stiffness and pain from multiple joints caused by non-erosive polyarthritis, and the presence of autoantibodies directed to nuclear antigens, called antinuclear antibodies (ANA). These ANA are also commonly present in both human and canine SLE and other systemic rheumatic diseases^11,14,15^. Several genetic risk factors for IMRD have been identified^16,17^. Interestingly, particular subgroups of ANA are associated with different, but overlapping sets of genes in dogs^18^.

Autoantibodies are often directed at proteins that are highly conserved between species^19^. The standard test for detecting canine ANA – indirect immunofluorescence microscopy (IIF) – uses cells of human origin (HEp-2) as substrate^20^. In a previous study, it could be shown that several autoantibodies with important roles in human disease were also present in dogs^21^. Thus, the autoantibody targets in human and canine disease frequently overlap, although there are some autoantibodies that are species-specific^22^.

Developments in proteomic technologies have opened for new ways to study autoimmune diseases. Human protein arrays, which contain thousands of full-length proteins, have been successfully used to identify novel autoantibody targets in human autoimmune diseases^23–26^. The main aim of the present study was to use protein arrays to identify new autoantigens in canine autoimmune disease.

## Results

### ILF2 is an autoantigen in ANA positive dogs with IMRD

**Figure 1:**
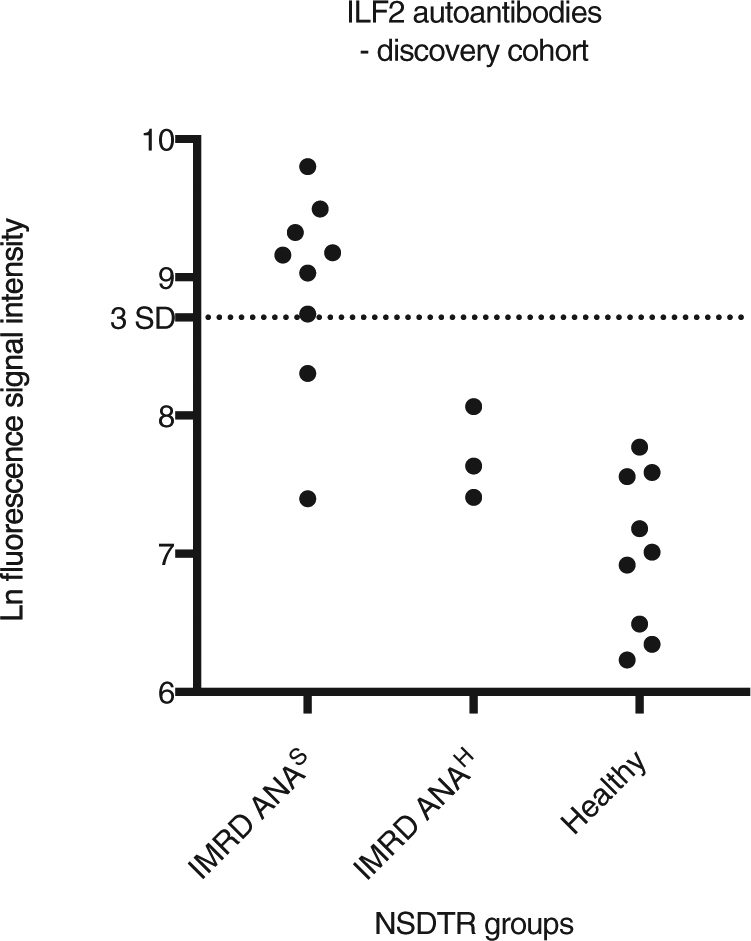
Identification of ILF2 as a potential autoantigen in canine IMRD. ILF2 was identified as a potential autoantigen in canine immune-mediated rheumatic disease (IMRD) by a protein array screen of 12 IMRD patients and 9 healthy controls. All of the dogs were of the breed Nova Scotia duck tolling retriever (NSDTR). Log(ln)-transformed fluorescence signal intensities are the mean of protein duplicates. The cutoff value was calculated from healthy controls as the mean + 3 SD (ln-transformed data). IMRD ANA^S^, IMRD patients with speckled antinuclear antibodies (ANA) pattern. IMRD ANA^H^, IMRD patients with homogenous ANA pattern.

A panel of approximately 17,000 human full-length proteins was used to identify autoantigens in ANA positive dogs with IMRD. The workflow of the study is presented in Supplementary Fig. 1. In the discovery phase, we focused our studies on two subgroups of these IMRD patients with different ANA patterns on IIF: speckled (ANA^S^) and homogenous (ANA^H^). Sera from nine canine patients with ANA^S^, three with ANA^H^, and nine healthy controls were used to screen the protein arrays (Supplementary Table 1). All the ANA^S^ sera had tested negative for reactivity to 18 known autoantigens, meaning that a broad screen would have the potential to uncover previously undetected autoantigens (Supplementary Fig. 2). To select for patient-associated autoantibody signals, we used a cutoff value, based on the healthy controls, of the mean + 3 SD (log-transformed data) for each antigen. Low level signals (below the mean + 3 SD for each sample) were removed. This approach identified 90 antigens with increased autoantibody signals in at least one IMRD patient. To search for common antigen-targets, we excluded antigens with autoantibody signals present in only one of the patients. This identified seven autoantigens (ILF2, FAM134B, CCDC97, ETFB, PAIP2, CTDP1 and NUBP1), of which ILF2 was the most promising candidate autoantigen. ILF2 autoantibodies were detected in seven of the nine IMRD patients with ANA^S^, but were absent in all patients with ANA^H^ and the healthy controls (Fig. 1). In comparison, signals to the second most common target, FAM134B, were present in only three of the 12 IMRD patients.

**Figure 2:**
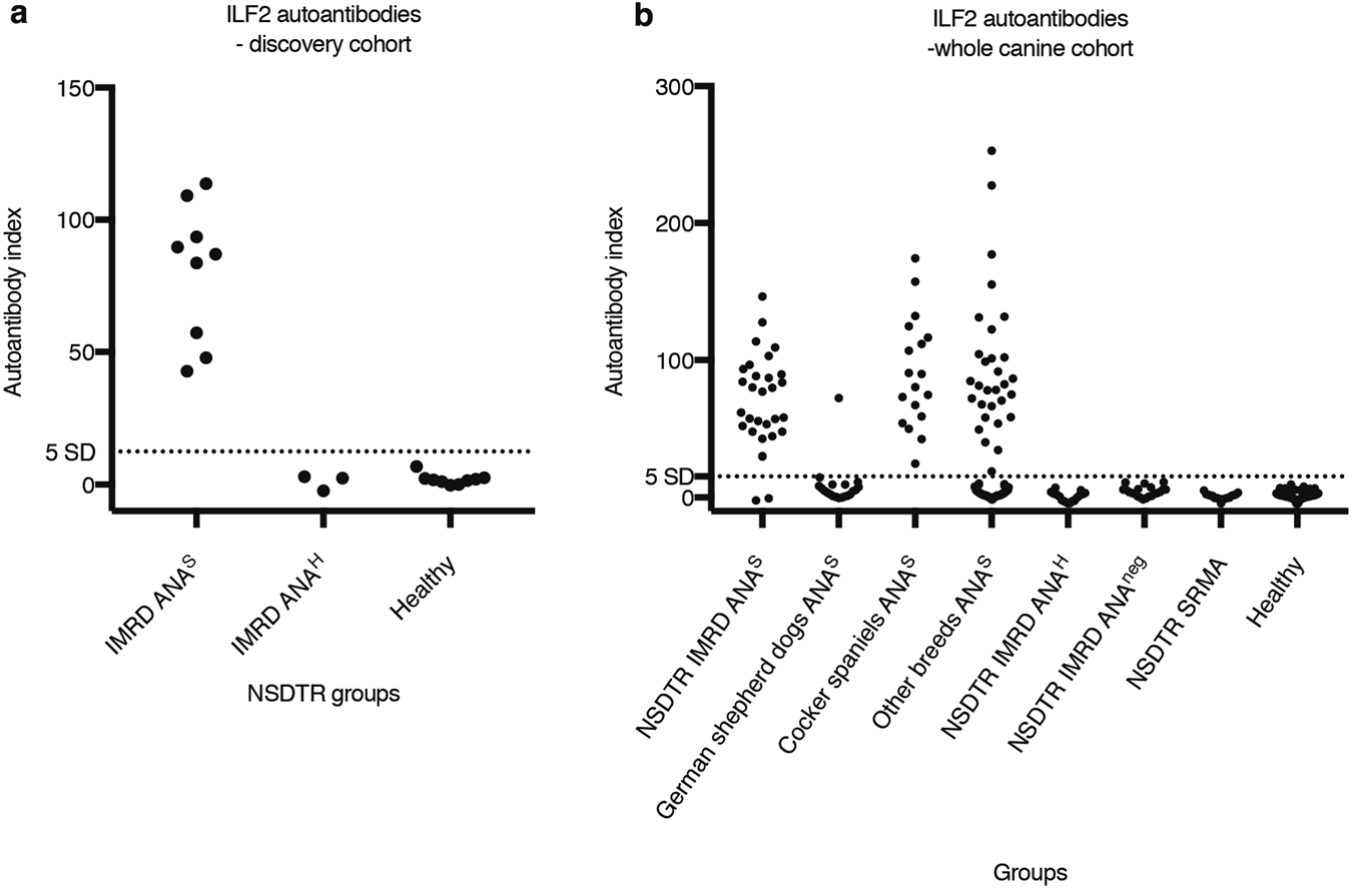
Validation of ILF2 with an independent method. Radio-ligand binding assay was used to validate ILF2 as an autoantigen in the discovery cohort (**a**) and in an extended cohort. (**b**) represent discovery and extended cohort together. All of the patients with immune-mediated rheumatic disease (IMRD) were of the breed Nova Scotia duck tolling retriever (NSDTR). The cutoff value was calculated from healthy controls as the mean + 5 SD. All samples were analysed in duplicate. Autoantibody index = (sample value mean − negative control)/(positive control − negative control) * 100. IMRD ANA^S^, IMRD patients with speckled antinuclear antibodies (ANA) pattern IMRD. ANA^H^, IMRD patients with homogenous ANA pattern IMRD. ANA^neg,^ IMRD patients without ANA. SRMA, steroid-responsive meningitis-arteritis.

We next sought to validate ILF2 as a *bona fide* autoantigen using an independent method. Recombinant radio-labelled ILF2 was expressed *in vitro* and immunoprecipitated with patient and control sera. The radio-ligand binding assay (RLBA) confirmed the results of the protein array screen. It identified all of the sera from IMRD ANA^S^ patients (i.e., the discovery cohort) as ILF2-positive and all the patients with ANA^H^ and healthy controls as negative (cutoff = mean + 5 SD of healthy controls) (Fig. 2a). Because it appeared that ILF2 was a common autoantigen in the IMRD ANA^S^ patients, we screened an extended cohort of NSDTRs with IMRD ANA^S^ and identified ILF2 autoantibodies in 18 of the 20 available sera. When the discovery and extended cohorts were considered together, ILF2 autoantibodies were present in 27 of 29 (93.1%) ANA^S^ sera from NSDTRs with IMRD (Fig. 2b). In the extended cohort we also screened 121 ANA^S^ sera from 43 other breeds where the dog was suspected to have an autoimmune disease. In these samples, 49 (40.5%) of the sera were ILF2-positive (Fig. 2b). A marked difference was obvious between breeds in the frequency of ILF2 autoantibodies when comparing two of the most prevalent breeds in the study (Supplementary Table 2). While all 18 cocker spaniels had ILF2 autoantibodies, only one of the 20 German shepherd dogs was ILF2-positive. Furthermore, ILF2 autoantibodies were exclusively found in ANA^S^ sera; they were not present in sera from the 84 healthy controls or in the 57 patients with IMRD ANA^H^, IMRD without ANA, or with steroid-responsive meningitis-arteritis (Fig. 2b).

### Autoantibodies to ILF2 and ILF3 are found in association

**Figure 3:**
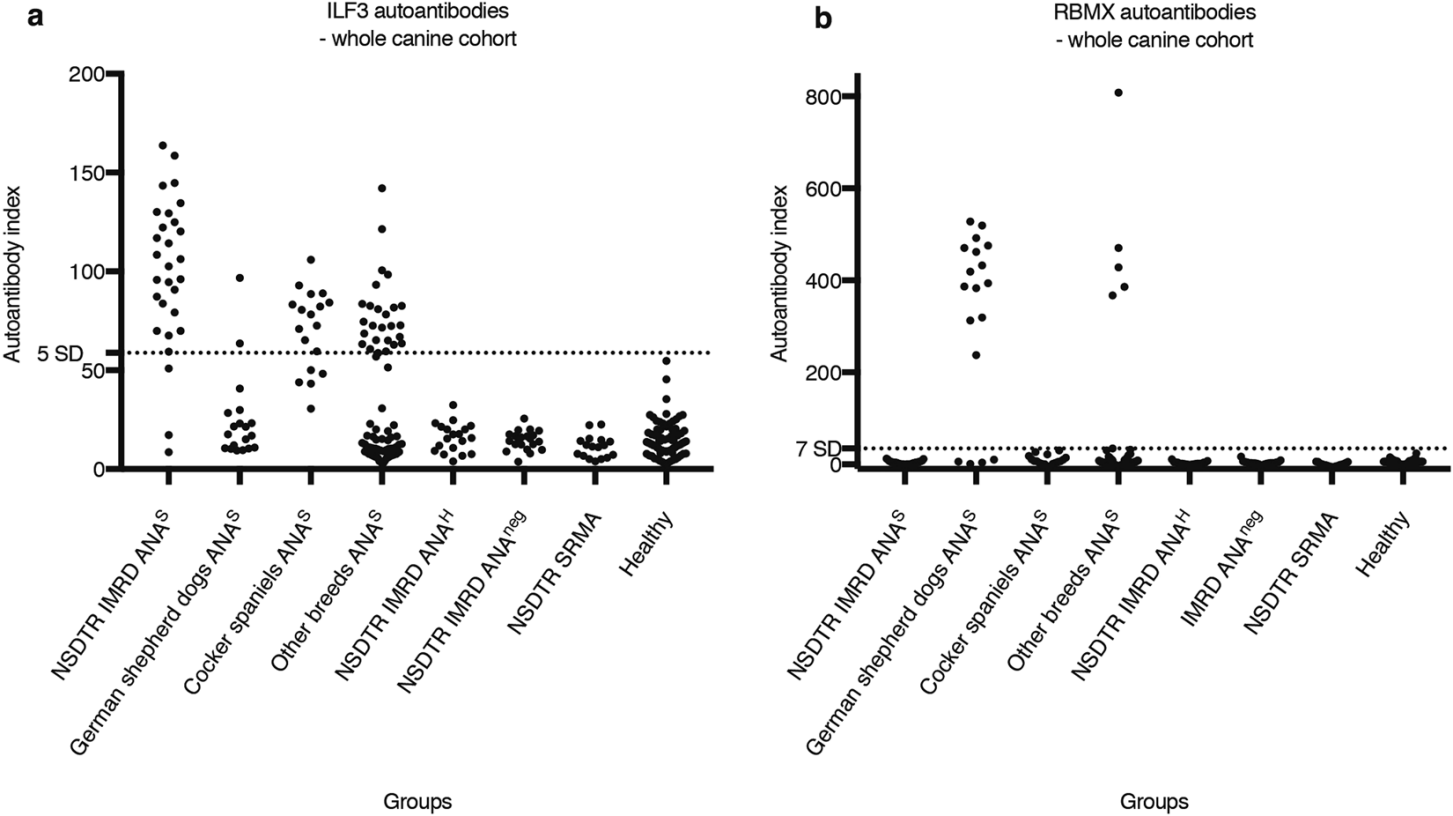
Screening the canine cohort for ILF3 and RBMX autoantibodies. A radio-ligand binding assay was used to screen for ILF3 (**a**) and RBMX (**b**) autoantibodies in dogs. The cutoff value for ILF3 was calculated from healthy controls as the mean + 5 SD. For RBMX, the mean + 7 SD was used as cutoff value because of a clear separation of negative and positive samples. All samples were analysed in duplicate. Autoantibody index = (sample value mean − negative control)/(positive control − negative control) * 100. NSDTR, Nova Scotia duck tolling retriever. IMRD ANA^S^, IMRD patients with speckled antinuclear antibodies (ANA) pattern. IMRD ANA^H^, IMRD patients with homogenous ANA pattern. IMRD ANA^neg,^ IMRD patients without antinuclear antibodies. SRMA, steroid-responsive meningitis-arteritis.

The ILF2 protein is expressed predominantly in the nucleus as a heterodimer complex with ILF3^27^. Because of this association, we asked whether ILF3 might also be a target of autoantibodies in the IMRD patients. RLBA was used to screen the canine cohort for ILF3 autoantibodies. As with the ILF2 autoantibodies, ILF3 autoantibodies were present only in ANA^S^ sera – in 26 of 29 (89.7%) of the NSDTRs with IMRD and in 41 of 120 (34.2%) of the ANA^S^ from dogs of other breeds, including German shepherd dogs and cocker spaniels (Fig. 3a). All cocker spaniel sera (n = 18) were above baseline, with 13 sera testing positive above the cutoff value.

**Figure 4:**
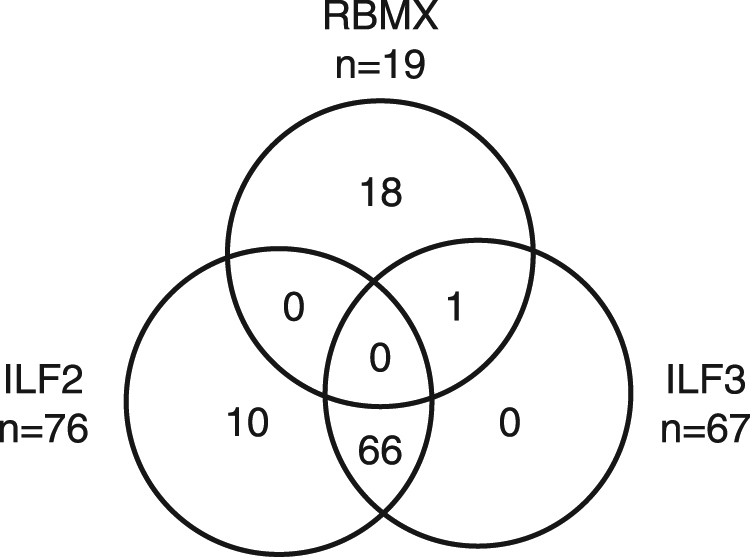
Reactivity to ILF2, ILF3 and RBMX. The number of positive samples to ILF2, ILF3 and RBMX is presented in each field.

ILF2 and ILF3 proteins are also known to form complexes with other proteins, including RNA-binding motif protein, X chromosome – RBMX (also known as hnRNP G)^28,29^. Interestingly, RBMX has previously been described as an autoantigen in dogs with lupus-like disease and ANA^S^^22,30^. To better understand the relation between RBMX autoantibodies and those of ILF2 or ILF3, we screened the total canine cohort (discovery and extended) for RBMX autoantibodies. Autoantibodies to RBMX were detected in 14 of 18 ANA^S^ sera from the German shepherd dogs, and in five of the 83 ANA^S^ serum samples from other breeds (Fig. 3b). Of these five RBMX-positive dogs, four were cross-breeds and one dog of unknown breed. Only one of the 19 RBMX-positive dogs also had autoantibodies to ILF3, while no dog was positive for both RBMX and ILF2 (Fig. 4).

In total, 289 samples were analysed for autoantibodies to the three antigens: 66 were positive for both ILF2 and ILF3 and 10 were exclusively ILF2-positive (Fig. 4). None of the sera were positive for ILF3 only, or for all three antigens. Of the 148 ANA^S^ samples analysed for autoantibodies to all three antigens, 95 (64.2%) were positive for at least one antigen and 77 (52.0%) for ILF2 and/or ILF3. Because ILF2 and ILF3 autoantibodies often co-existed in patients, we investigated the possibility of cross-reactivity using an immune competition approach. Labelled *in vitro* translated proteins at a constant concentration (1 U ≈ 30,000 cpm), and unlabelled proteins at increasing concentrations (1 to 32 U), were allowed to react with ILF2- and ILF3-positive sera. The ILF2 immunoreactivity was not reduced by unlabelled ILF3, and vice versa, *i*.*e*., ILF3 immunoreactivity was not reduced or eliminated by unlabelled ILF2 (Supplementary Fig. 3). As expected, the ILF2 immune reactivity could be eliminated by increasing concentrations of unlabelled ILF2 and that of ILF3 by increasing concentrations of unlabelled ILF3.

**Figure 5:**
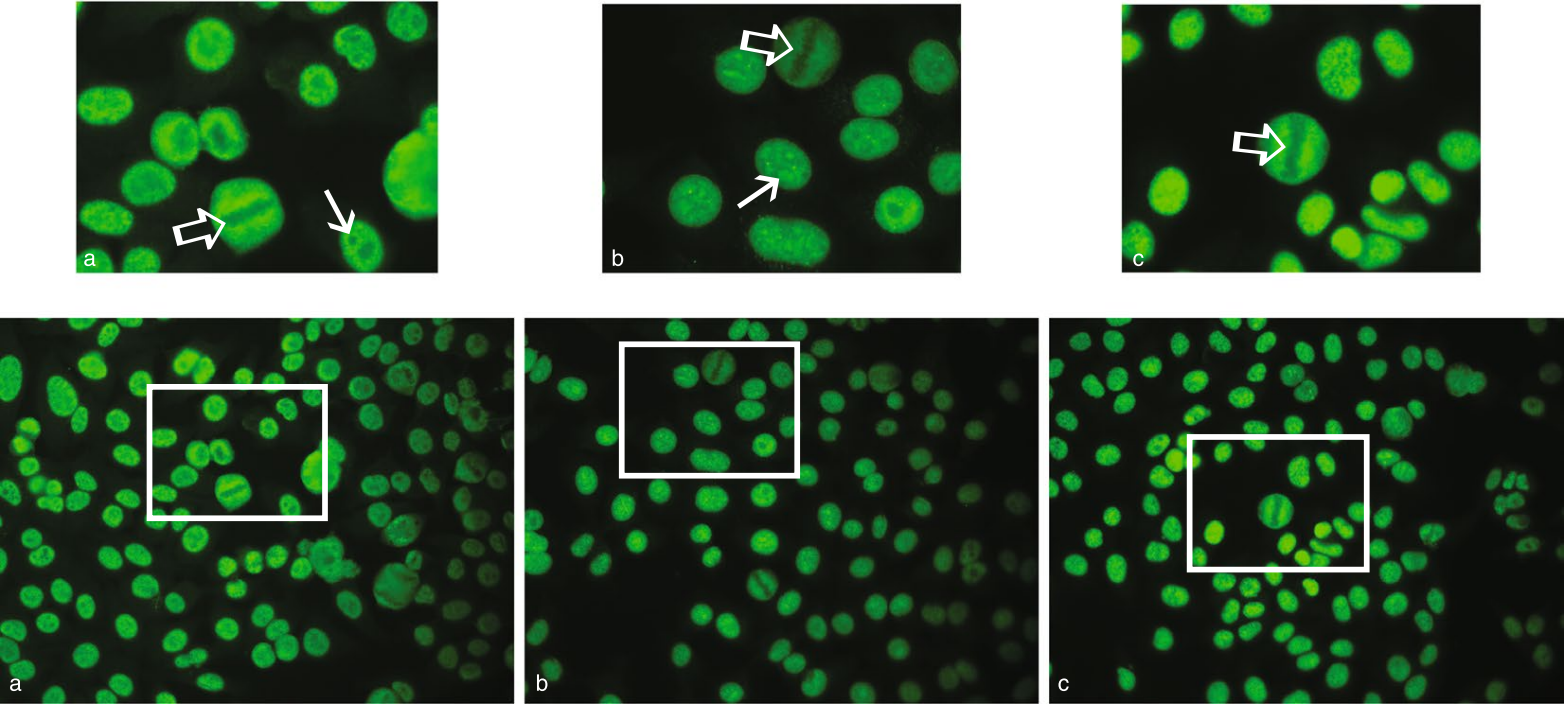
Sera from ILF2- and ILF3-positive patients and commercial ILF2 and ILF3 antibodies display a speckled ANA pattern. Indirect immunofluorescence microscopy images of HEp-2 cells incubated with sera from an ILF2- and ILF3-positive IMRD patient (**a**), a commercial polyclonal ILF2 antibody (**b**); and a commercial polyclonal ILF3 antibody (**c**). (**a**) In the nucleoplasm, fine tiny speckles can be observed and mitotic cells have unstained chromatin mass (⇨). The nucleoli are not stained (→). (**b,c)** In the nucleoplasm, fine tiny speckles can be observed and mitotic cells have unstained chromatin mass (⇨). Nucleoli are stained in many of the cells (→) but not all.

To illustrate the type of staining pattern of ILF2- and ILF3-positive sera, we stained HEp-2 cells with the autoantibody-positive canine sera and had the images evaluated by an experienced interpreter. Incubation with the ILF2- and ILF3-positive sera showed tiny speckles across all nucleoplasm in which neither the chromatin mass of mitotic cells nor the nucleoli took up the stain (Fig. 5a). This was interpreted as a fine-speckled ANA pattern, AC-4^31^. We also performed IIF with commercial ILF2 and ILF3 antibodies, which displayed a similar fine speckled staining pattern (AC-4); here, however, some but not all nucleoli were stained (Fig. 5b and c).

### Screening of human patients with SLE or Sjögren’s syndrome for ILF2 and ILF3 autoantibodies

To assess the presence of ILF2 and ILF3 autoantibodies in human disease, we screened human sera from patients with SLE, Sjögren’s syndrome or systemic sclerosis (scleroderma) (Supplementary Fig. 4). Autoantibodies to ILF2 were detected in one Sjögren’s syndrome patient, and at a level just above cutoff in one SLE patient. Similarly, ILF3 autoantibodies were detected in a different Sjögren’s syndrome patient and in two other SLE patients, but again close to the cutoff level. All healthy controls were negative for both ILF2 and ILF3 autoantibodies.

## Discussion

Here we identified ILF2 and ILF3 as major autoantigens in canine systemic autoimmune disease. These two proteins are expressed as heterodimers in many tissues where they function as transcription factors crucial for expression of, for example, interleukin-2 and interleukin-13^27,32–35^. Autoantibodies to ILF2 and ILF3 have previously been described in mice with induced lupus and as a rare finding in humans with autoimmune disease^36,37^. This highlights the fact that autoantibody targets are often conserved across species. The methods to identify the autoantibodies differ between ours and the previous studies.

In dogs, we found the autoantibodies to ILF2 and ILF3 exclusively in ANA^S^ sera. The speckled ANA pattern is the most frequently observed ANA pattern in dogs^8,20^, and is also a major pattern observed in humans, where it is associated with disorders such as Sjögren’s syndrome, SLE, and systemic sclerosis^31^. Autoantibodies to ILF2 and/or ILF3 were found in 52.0% of the 148 canine ANA^S^ sera analysed in this study. In comparison, autoantibodies to RBMX, which has previously been described as an important autoantigen in dogs with ANA^S^, were found in only 12.8% of the ANA^S^ sera. Many dogs had autoantibodies to both ILF2 and ILF3, and our results from the immune competition suggest that this is not due to cross-reactivity but rather to specific binding.

To our knowledge, this is the first report of the autoantigens ILF2 and ILF3 in dogs. One explanation for why these autoantigens have not been previously reported could be that ILF2 (∼45 kDa) and RBMX (∼43 kDa) are of similar size. In a canine study by Welin Henriksson *et al*.^38^, immunoblot reactivity to a ∼43 kDa protein was frequently found in ANA^S^ sera; the antigen was assumed to be RBMX based on the molecular mass. Similarly, Monier *et al*.^15^ describe autoantibodies to a ∼43 kDa protein in dogs with lupus-like disease and ANA^S^. In the light of the present study, it seems reasonable to assume, that at least some, if not most, of these samples, actually contained ILF2 autoantibodies. Soulard *et al*.^39^ also describe autoantibodies to a set of proteins of 45, 95, and 110 kDa in dogs suffering from autoimmune disorders. These unidentified antigens, described to be present in the nucleoplasm and nucleoli of HEp-2 and HeLA cells, could have been ILF2 (45 kDa) and ILF3 (90 and 110 kDa^40^). Even though ILF2 and ILF3 are expressed in nucleoli^41,42^, autoantibodies in this area of the cell nucleus could not be detected by IIF using serum antibodies. One explanation for this could be that the concentration of serum antibodies was lower than that of the commercial antibodies.

Intriguingly, there was a strong breed difference regarding ILF2, ILF3 and RBMX autoantibodies. While ILF2 and ILF3 were common antigen targets in NSDTRs and cocker spaniels, RBMX autoantibodies were exclusively found in German shepherd dogs, cross-breeds, and in one dog of unknown breed. This is in agreement with previous studies where RBMX has been described as an antigen mainly in German shepherd dogs with SLE or lupus-like disease^43^. Interestingly, in a study by Satoh *et al*.^44^, only two of 11 tested mouse strains developed ILF2 and ILF3 autoantibodies upon treatment with pristane, a chemical that triggers lupus-like disease. In contrast, most of their mouse strains developed autoantibodies to well-known SLE autoantigens, like RNP and Sm, upon treatment with pristane. Thus, it seems like the genetic background is important for the development of autoantibodies to ILF2 and ILF3.

While ILF2 and ILF3 autoantibodies are a common finding in dogs with autoimmune diseases, our screening of human sera is in line with previous reports that ILF2 and ILF3 autoantibodies are only occasionally present in humans. Satoh *et al*.^36^ identified ILF2 and ILF3 autoantibodies in only two of 1200 human sera. The study does not state if the investigated cohort included healthy controls or which different autoimmune disorders were assessed. Previously, Labrador *et al*.^45^ published a report of autoantibodies to a set of unidentified proteins in three patients with rheumatoid arthritis and features of systemic sclerosis. Satoh *et al*.^36^ later identified these patients as positive for ILF2 and ILF3 autoantibodies. Further investigation of larger cohorts of human patients with different and clearly defined autoimmune manifestations, is warranted to better understand the role of ILF2 and ILF3 autoantibodies in human disease. Similarly, the clinical signs of ILF2 and ILF3 autoantibody-positive dogs need to be thoroughly evaluated, to determine the diagnostic value of these autoantibodies as biomarkers. We could not find any associations between ILF2 and ILF3 autoantibodies and the clinical information that was available from the medical records of the NSDTRs with IMRD. It is possible that these autoantibodies could be used as a clinical biomarkers in other breeds, but there was no clinical information available in our study for these other breeds.

Several autoantigens have been previously identified in dogs by different approaches^9,21,22,38,46–50^. Here, we report the use of human protein arrays for the screening of canine autoantigens. To our knowledge, this type of discovery screening for autoantigens has not previously been applied to dogs and it allowed us to discover two new autoantigens, ILF2 and ILF3. The method is developed for screening a large number of antigen targets, but it has also some limitations. An antigen might fail to produce a signal due to technical factors, rather than absence of autoantibodies in the sample^26^. Furthermore, the proteins on the array are recombinant human ones, and the degree of protein conservation between humans and dogs varies. Therefore, negative results should not be interpreted as truly autoantibody-negative as autoantibodies usually reacts with conformationally sensitive epitopes that may be destroyed when presented on a surface^26^. For example, in our discovery screening, ILF3 was present on the array, but failed to generate an elevated signal in any of the samples that were ILF3-positive on RLBA. A positive result should also be interpreted with caution, since many initial reports of biomarkers cannot be reproduced^51^, and should be validated with an independent method. Here we used RLBA for validation, which presents the autoantigens in solution, and also allowed screening a larger number of samples. This would not have been possible in the discovery phase due to the substantial costs for the human protein arrays. In our experience, the RLBA is a sentitive and specific method for autoantibody detection, but we cannot rule out false negative samples, since different epitopes could potentially be present *in vivo*.

To conclude, by screening for a large number of autoantigen targets in a small, selected canine cohort, we identified ILF2, for the first time, as a canine autoantigen. By expanding the screen to include a larger number of canine samples from dogs of multiple breeds, we found that autoantibodies to ILF2, and the associated protein ILF3, were frequently present in canine ANA^S^ sera. Furthermore, ILF2 and ILF3 autoantibodies were present in human patients with autoimmune disease, but as a rare finding. These autoantibodies have the potential to be used as diagnostic biomarkers in both species, but how to use this information needs to be further studied. We also showed that human protein arrays can be successfully used to identify canine autoantigens. The use of these arrays should lead to more autoantigen discoveries in veterinary medicine.

## Materials and Methods

### Canine subjects

The canine serum samples were collected between 2002 and 2014 and placed at −80 °C for long-term storage; some of the samples were temporarily stored at −20 °C before long-term storage. All experiment were performed in accordance with relevant guidelines and regulations and ethical permission was obtained from the Uppsala Animal Ethical Committee and Swedish Board of Agriculture (C417/12, C418/12 and C15/16).

All of the IMRD patients were NSDTRs. The dogs diagnosed with IMRD displayed musculoskeletal signs with lameness, stiffness and pain from two or more joints on manipulation. The signs had been apparent for at least 14 days prior to sampling and were the main reason for the veterinary visit. The diagnostic tests to rule out other diseases varied between the dogs, but no other diseases than IMRD were suspected as the main cause of the clinical signs. All suspected IMRD cases were tested for ANA with IIF, as previously described^20^. Positive ANA and response to glucocorticoid treatment strengthened the diagnosis. Some of the dogs were under treatment with either non-steroid anti-inflammatory drugs or glucocorticoids at the time of sampling. Control dogs were considered healthy at the time of sampling based on clinical examination and owner interview.

### Discovery cohort

In the protein array screen, sera from 21 NSDTRs were included: twelve with IMRD and nine healthy controls (Supplementary Table 1). The sera were selected from a larger cohort of NSDTRs that had been clinically characterised with respect to immune disease. The sera had also been tested for ANA with IIF. The twelve IMRD sera had also been tested for autoantibodies to 18 antigens associated with human autoimmune or rheumatic disorders using ELISA and line blot, as described previously^21^. All twelve IMRD sera tested positive on the IIF-ANA test – nine with a speckled pattern (ANA^S^), and three with a homogenous pattern (ANA^H^) (Supplementary Fig. 2). The nine ANA^S^ sera and two of the ANA^H^ sera were negative for all 18 tested antigens, while one of the ANA^H^ sera showed both nucleosome and dsDNA reactivity with ELISA, and weak SS-A/Ro-52 reactivity with line blot. The healthy controls were all ANA negative.

### Validation cohort

In the validation of ILF2, and in the ILF3 and RBMX screening, the 21 serum samples from the protein array screen were tested together with 270 additional serum samples (but 269 for ILF3 and 268 for RBMX, due to lack of sera), (Supplementary Table 2). In total, 29 ANA^S^ sera from IMRD NSDTRs were screened for ILF2, ILF3 and RBMX autoantibodies. We also included 121 ANA^S^ sera (120 for ILF3, 119 for RBMX) from a previous study^21^ consisting of sera from dogs of 43 different breeds. These included 20 German shepherd dogs (19 for ILF3, 18 for RBMX), 18 English and American cocker spaniels, 11 dogs of mixed breed, 7 golden retrievers, and 65 of other or unknown breeds. Clinical information was unavailable for these 121 dogs, but sera had been sent to our laboratory for routine ANA testing, presumably because of suspicion of autoimmune disease.

Included as healthy controls were 84 dogs: 33 NSDTRs, 19 German shepherd dogs, and 32 of other breeds (13 border collies, 9 beagles, 6 labradors, 3 Australian shepherds, and 1 Boston terrier). We also included 57 disease controls consisting of NSDTRs with IMRD and ANA^H^, IMRD without ANA, or steroid-responsive meningitis-arteritis, another immune-mediated disease described in the breed^52^. One of the dogs with steroid-responsive meningitis-arteritis was also included in the IMRD ANA^S^ group. This dog was diagnosed with steroid-responsive meningitis-arteritis at an age of 10 months and with IMRD at two years of age. Samples were taken 16 months apart for this dog.

### Human subjects

Patients with SLE or Sjögren’s syndrome were included from the Rheumatology Clinic at Uppsala University Hospital, Sweden. As controls, sera from healthy blood donors from the Uppsala Bioresource^53^, and from previous studies^23,24^, were included. Sera were analysed for ILF2 (91 Sjögren’s syndrome, 38 SLE, 70 systemic sclerosis and 182 blood donors) and ILF3 (107 Sjögren’s syndrome, 38 SLE and 88 blood donors) autoantibodies with RLBA. The study was approved by the local ethic committees in Uppsala and Stockholm (97358, 2006/217, 00-399, 2016/2553-31/2) and was performed in accordance with the Declaration of Helsinki and to relevant regulations. All participants gave their informed consent.

### Indirect immunofluorescence microscopy

HEp-2 cells (RB-2100, Immunoconcepts) were incubated with rabbit polyclonal anti-ILF2 (HPA0007484, Atlas Antibodies) at a 1:100 dilution (2 μg/ml) and rabbit polyclonal anti-ILF3 (HPA001897, Atlas Antibodies) at a 1:50 dilution (2 μg/ml) for 30 min. After 5 min of washing in PBS, cells were incubated with Alexa Fluor 488 conjugated goat anti-rabbit IgG (A-11034, Thermofisher Scientific) at a 1:200 dilution (10 μg/μl) for 30 min. The slides were washed with PBS and covered with coverslips before immunofluorescence imaging. HEp-2 cells were also incubated with canine patient sera at a 1:100 dilution followed by incubation with FITC conjugated rabbit anti-dog IgG at 1:100 dilution (F7884, Sigma-Aldrich). Images were taken using a Zeiss LSM 510 microscopy with the 40X objective.

### Protein array screening

Protein arrays (HuProt^TM^ v2 Human Proteome Arrays, CDI laboratories) were incubated with sera from dogs with IMRD and from healthy controls. The arrays were first incubated with blocking buffer (3% BSA in PBS-T) for 1 h, and thereafter with 5 ml diluted serum (1:200 in PBS-T (0.1%), 3% BSA and 5% milk powder) for 1 h. The arrays were then washed with PBS-T for 5 × 5 min. Next, arrays were incubated with Alexa Fluor 647 conjugated rabbit anti-dog IgG (304-605-00, Jackson ImmunoResearch) at a concentration of 0.04 μg/ml for 1 h, followed by washing with PBS-T for 5 × 5 min. Arrays were then incubated with anti-GST (Goat Anti-GST Dylight 550, 11254, Cayman Chemicals) at a concentration of 0.14 μg/ml for 1 h. The arrays were thereafter washed for 4 × 5 min with PBS-T followed by 1 × 5 min with PBS before being dipped 5 times in deionized water and centrifuged dry. Incubation and washing steps were performed in a four-chamber tray set at 50 rpm rotation at room temperature and arrays were covered from light starting with the anti-dog IgG incubation step. The arrays were scanned using a Agilent G2505C microarray scanner (Agilent Technologies) and GenePix Pro 5.1.0.19 microarray software was used for alignment and data acquisition.

### Radio-ligand binding assay

A RLBA was used to screen sera for autoantibodies to ILF2, ILF3 and RBMX. Human ILF3 cDNA in a pCMV6-Entry vector (RC214999, Origene) was used for *in vitro* transcription and translation of ILF3. Human ILF2 cDNA (RC201751, Origene) and human RBMX cDNA (RC 200777, Origene) were first subcloned into a pTNT^TM^ vector (Promega, L5610) to enable *in vitro* transcription and translation. Sanger sequencing confirmed the respective clones as ILF2, ILF3 and RBMX.

*In vitro* transcription and translation was performed in the presence of ^35^S-methionine according to the manufacturer’s protocol (Promega TNT Systems). Immunoprecipitation was performed in 96-well titration plates overnight at 4 °C at 300 rpm rotation. A positive standard (anti-ILF2 antibody, HPA007484; anti-ILF3 antibody, HPA0018979; anti-RBMX, HPA057707, Atlas Antibodies) and a negative standard of 4% BSA were included in each plate. To each well, 2.5 μl of sera and radiolabelled protein (~30,000 cpm) were added. All samples were analysed in duplicate. After incubation, the immune reaction was transferred to filter plates and serum antibodies were immobilised to Protein A Sepharose (nProtein A sepharose 4 Fast Flow, GE Healthcare) during 45 min of incubation at 4 °C at 300 rpm rotation. The plates were then washed 10 times with washing buffer (150 mM NaCl, 20 mM Tris/HCl, 0.15% Tween 20 and 0.1% BSA) and dried. Scintillation solution (Optiphase supermix, Perkin Elmer) was added to all the wells (70 μl/well) and radioactivity was measured in a microbeta counter (1450 MicroBeta TriLux, Wallac). Autoantibody index values were calculated according to the equation: Index = ((sample value mean − negative control)/(positive control − negative control)) × 100.

### Immune competition assay

Serum from an ILF2- and ILF3 autoantibody-positive dog was selected and immunoreactivity was tested by RLBA in a dilution series from 1:20 to 1:40,960. The highest dilution that gave a clear positive result was 1:1,280 and was used for the experiments. Radioactive (labelled) and non-radioactive protein (unlabelled) were produced in parallel by *in vitro* transcription and translation. One unit of protein was represented by 30,000 cpm. Sera were allowed to react with 1 U of labelled protein and 0,1,2,4,8,16 and 32 U of unlabelled protein; reactivity was measured in the same way as described for the RLBA.

### Statistical analyses

Statistical analyses of protein array data were performed on ln-transformed signal intensities. To identify antigen signals in the IMRD patients, cutoff values for each antigen were calculated from healthy controls as the mean + 3 SD. Signal-to-noise filtration was then performed for every sample by removing all signals below the mean + 3 SD for each sample (global filtration).

In the radio-ligand binding assay, cutoff values for ILF2 and ILF3 were calculated from healthy controls as the mean + 5 SD. For RBMX, there was a clear separation of samples that appeared negative and positive, and therefore a higher cutoff of the mean + 7 SD was applied.

All statistical calculations were performed in software R v.3.0 and Excel v.15.32.

### Data availability

All relevant data are available from the authors upon request.

## Electronic supplementary material

Supplementary information

## References

[1] Gershwin LJ (2007). Veterinary autoimmunity: autoimmune diseases in domestic animals. Ann. N. Y. Acad. Sci..

[2] Elkon K, Casali P (2008). Nature and functions of autoantibodies. Nat. Clin. Pract. Rheumatol..

[3] Dawoodji A (2014). High frequency of cytolytic 21-hydroxylase-specific CD8+) T cells in autoimmune Addison’s disease patients. J. Immunol..

[4] Tan EM (2012). Autoantibodies autoimmune disease, and the birth of immune diagnostics. J Clin Invest.

[5] Scofield RH (2004). Autoantibodies as predictors of disease. Lancet.

[6] von Mühlen CA, Tan EM (1995). Autoantibodies in the diagnosis of systemic rheumatic diseases. Semin. Arthritis Rheum..

[7] Lewis RM, Schwartz R, Henry WB (1965). Canine systemic lupus erythematosus. Blood.

[8] Hansson-Hamlin H, Lilliehöök I, Trowald-Wigh G (2006). Subgroups of canine antinuclear antibodies in relation to laboratory and clinical findings in immune-mediated disease. Vet Clin Pathol..

[9] Fournel C (1992). Canine systemic lupus erythematosus. I: A study of 75 cases. Lupus.

[10] Karlsson EK, Lindblad-Toh K (2008). Leader of the pack: gene mapping in dogs and other model organisms. Nat Rev Genet.

[11] Tan, E. M. *et al*. The1982 revised criteria for the classification of systemic lupus-erythematosus. *Arthritis Rheum*. **25**, 10.1002/art.1780251101 (1982). 10.1002/art.1780251101 7138600

[12] Ettinger, S. J., Feldman, E. C. & Coté, E. *Textbook of Veterinary Internal Medicine*. 8 edn, Vol. 1, 875 (Elsevier, St.Louis, Missouri, 2017).

[13] Hansson-Hamlin, H. & Lilliehöök, I. A possible systemic rheumatic disorder in the Nova Scotia duck tolling retriever. *Acta Vet*. *Scand*. **51**:**16**, 10.1186/1751-0147-51-16 (2009). 10.1186/1751-0147-51-16 PMC2667523 19331658

[14] Bennett D, Kirkham D (1987). The laboratory identification of serum antinuclear antibody in the dog. J. Comp. Pathol..

[15] Monier JC (1992). Canine systemic lupus-erythematosus. 2. Antinuclear antibodies. Lupus.

[16] Wilbe, M. *et al*. Genome-wide association mapping identifies multiple loci for a canine SLE-related disease complex. *Nat*. *Genet*. **42**, 10.1038/ng.525 (2010). 10.1038/ng.525 20101241

[17] Wilbe M (2009). MHC class II polymorphism is associated with a canine SLE-related disease complex. Immunogenetics.

[18] Wilbe, M. *et al*. Multiple changes of gene expression and function reveal genomic and phenotypic complexity in SLE-like disease. *PLoS Genet* **11**, 10.1371/journal.pgen.1005248 (2015). 10.1371/journal.pgen.1005248 PMC4461293 26057447

[19] Utz PJ, Gensler TJ, Anderson P (2000). Death, autoantigen modifications, and tolerance. Arthritis Res..

[20] Hansson H, Trowald-Wigh G, Karlsson-Parra A (1996). Detection of antinuclear antibodies by indirect immunofluorescence in dog sera: comparison of rat liver tissue and human epithelial-2 cells as antigenic substrate. J. Vet. Intern. Med..

[21] Bremer HD (2015). Identification of specific antinuclear antibodies in dogs using a line immunoassay and enzyme-linked immunosorbent assay. Vet. Immunol. Immunopathol..

[22] Soulard M (1991). A novel 43-kDa glycoprotein is detected in the nucleus of mammalian cells by autoantibodies from dogs with autoimmune disorders. Exp. Cell Res..

[23] Landegren N (2016). Proteome-wide survey of the autoimmune target repertoire in autoimmune polyendocrine syndrome type 1. Sci. Rep..

[24] Landegren N (2015). Transglutaminase 4 as a prostate autoantigen in male subfertility. Sci. Transl. Med..

[25] Price JV (2013). Protein microarray analysis reveals BAFF-binding autoantibodies in systemic lupus erythematosus. J. Clin. Invest..

[26] Duarte JG, Blackburn JM (2017). Advances in the development of human protein microarrays. Expert Rev Proteomics.

[27] Corthesy B, Kao PN (1994). Purification by DNA Affinity-Chromatography of 2 Polypeptides That Contact the Nf-at DNA-Binding Site in the Interleukin-2 Promoter. J. Biol. Chem..

[28] Ting NSY, Kao PN, Chan DW, Lintott LG, Lees-Miller SP (1998). DNA-dependent protein kinase interacts with antigen receptor response element binding proteins NF90 and NF45. J. Biol. Chem..

[29] Guan DY (2008). Nuclear factor 45 (NF45) is a regulatory subunit of complexes with NF90/110 involved in mitotic control. Mol. Cell. Biol..

[30] Soulard M (1993). hnRNP G - sequence and characterization of a glycosylated RNA-binding protein. Nucleic Acids Res..

[31] Chan, E. K. L. *et al*. Report of the first international consensus on standardized nomenclature of antinuclear antibody HEp-2 cell patterns 2014–2015. *Front*. *Immunol*. **6**, doi:UNSP 412 10.3389/fimmu.2015.00412 (2015). 10.3389/fimmu.2015.00412 PMC4542633 26347739

[32] Kao PN (1994). Cloning and expression of cyclosporin A- and FK506-sensitive nuclear factor of activated T-cells: NF45 and NF90. J. Biol. Chem..

[33] Zhao GH, Shi LF, Qiu DM, Hu H, Kao PN (2005). NF45/ILF2 tissue expression, promoter analysis, and interleukin-2 transactivating function. Exp. Cell Res..

[34] Kiesler P (2010). NF45 and NF90 Regulate HS4-dependent Interleukin-13 Transcription in T Cells. J. Biol. Chem..

[35] Shi LF, Godfrey WR, Lin J, Zhao GH, Kao PN (2007). NF90 regulates inducible IL-2 gene expression in T cells. J. Exp. Med..

[36] Satoh M (1999). Autoantibodies define a family of proteins with conserved double-stranded RNA-binding domains as well as DNA binding activity. J. Biol. Chem..

[37] Kuroda Y (2006). Induction of lupus-related specific autoantibodies by non-specific inflammation caused by an intraperitoneal injection of n-hexadecane in BALB/c mice. Toxicology.

[38] Welin Henriksson E, Hansson H, Karlsson-Parra A, Pettersson I (1998). Autoantibody profiles in canine ANA-positive sera investigated by immunoblot and ELISA. Vet. Immunol. Immunopathol..

[39] Soulard M (1989). Nucleolar proteins identified in human cells as antigens by sera from dogs with autoimmune disorders. Exp. Cell Res..

[40] Saunders LR, Jurecic V, Barber GN (2001). The 90-and 110-kDa human NFAR proteins are translated from two differentially spliced mRNAs encoded on chromosome 19p13. Genomics.

[41] Jarboui MA, Wynne K, Elia G, Hall WW, Gautier VW (2011). Proteomic profiling of the human T-cell nucleolus. Mol. Immunol..

[42] Thul, P. J. *et al*. A subcellular map of the human proteome. *Science* **356**, 10.1126/science.aal3321 (2017). 10.1126/science.aal3321 28495876

[43] Soulard M, Della Valle V, Larsen CJ (2002). Autoimmune antibodies to hnRNPG protein in dogs with systemic lupus erythematosus: Epitope mapping of the antigen. J. Autoimmun..

[44] Satoh M (2000). Widespread susceptibility among inbred mouse strains to the induction of lupus autoantibodies by pristane. Clin. Exp. Immunol..

[45] Labrador M (1998). Antibodies against a novel nucleolar and cytoplasmic antigen (p105-p42) present in the sera of patients with a subset of rheumatoid arthritis (RA) with signs of scleroderma. Clin. Exp. Immunol..

[46] Hansson-Hamlin H, Rönnelid J (2010). Detection of antinuclear antibodies by the Inno-Lia ANA update test in canine systemic rheumatic disease. Vet. Clin. Pathol..

[47] Costa O, Fournel C, Lotchouang E, Monier JC, Fontaine M (1984). Specificities of antinuclear antibodies detected in dogs with systemic lupus-erythematosus. Vet. Immunol. Immunopathol..

[48] Monier JC (1980). Clinical and laboratory features of canine lupus syndromes. Arthritis Rheum..

[49] Monier JC (1988). Systemic lupus erythematosus in a colony of dogs. Am. J. Vet. Res..

[50] Monier JC (1978). Antibody to soluble nuclear antigens in dogs (German shepherd) with a lupus like syndrome. Dev. Comp. Immunol..

[51] Ioannidis JPA, Khoury MJ (2011). Improving Validation Practices in “Omics” Research. Science.

[52] Hansson-Hamlin H, Lilliehöök I (2013). Steroid-responsive meningitis-arteritis in Nova Scotia duck tolling retrievers. Vet. Rec..

[53] Berggren O (2015). IFN-alpha production by plasmacytoid dendritic cell associations with polymorphisms in gene loci related to autoimmune and inflammatory diseases. Hum. Mol. Genet..

