# Supplementary material for: ILF2 and ILF3 are autoantigens in canine systemic autoimmune disease
Source: Sci Rep. 2018 Mar 19;8:4852. doi: 10.1038/s41598-018-23034-w (PMC5859008; doi:10.1038/s41598-018-23034-w)

Supplementary information

ILF2 and ILF3 are autoantigens in canine systemic autoimmune disease

Hanna D Bremer, Nils Landegren, Ronald Sjöberg, Åsa Hallgren, Stefanie Renneker, Erik Lattwein, Dag Leonard, Maija-Leena Eloranta, Lars Rönnblom, Gunnel Nordmark, Peter Nilsson, Göran Andersson, Inger Lilliehöök, Kerstin Lindblad-Toh, Olle Kämpe, Helene Hansson-Hamlin

Supplementary Table 1. Age and sex of the dogs included in the discovery cohort

Status	Median age (range)	Female/Male	Number
NSDTRs with IMRD ANA^{S}	2.5 (1-4) y	3/6	9
NSDTRs with IMRD ANA^{H}	5 (4-6) y	2/1	3
Healthy control NSDTRs	2 (1-5) y	4/5	9

NSDTRs, Nova Scotia duck tolling retrievers
IMRD, immune-mediated rheumatic disease
ANA^{S}, antinuclear antibody (ANA) positive with a speckled pattern
ANA^{H}, ANA positive with a homogenous pattern
y, years

Supplementary Table 2. Age, sex and breed of all the dogs included in the study

Status	Median age (range)	Female/Male/Unknown	Number
NSDTRs with IMRD ANA^{S}	2 (1-6) y	17/12/0	29
ANA^{S} German shepherd dogs	5 (2-10) y	9/11/0	20$^{\#}$
ANA^{S} cocker spaniels*	3 (1-11) y	9/9/0	18
ANA^{S} other breeds	NA	39/43/1	83
NSDTRs with IMRD ANA^{H}	5 (2-12) y	16/3/0	19
NSDTRs with IMRD ANA^{neg}	7 (1-10) y	6/15/0	21
NSDTRs with SRMA	8 (8-23) m	10/7/0	17
Healthy controls	4 (1-13) y	52/32/0	84
**Total**		**158/132/1**	**291†**

NSDTRs, Nova Scotia duck tolling retrievers
IMRD, immune-mediated rheumatic disease
ANA^{S}, antinuclear antibody (ANA) positive with a speckled pattern
ANA^{H}, ANA positive with a homogenous pattern
ANA^{neg}, ANA negative
SRMA, steroid-responsive meningitis-arteritis
y, years
m, months
*Including English and American cocker spaniels
$^{\#}$ Due to lack of sera, only 19 samples were analysed for ILF3 autoantibodies and only 18 for RBMX autoantibodies
†291 sera from 290 dogs were analysed. One of the dogs is represented in both the IMRD ANA^{S} and the SRMA group.

Supplementary Fig. 1. Study design

Flow chart describing the study design to identify and validate ILF2 and ILF3 as autoantigens in canine and human autoimmune disease. Sera from Nova Scotia duck tolling retrievers with immune-mediated rheumatic disease (IMRD) and without, *i.e.*, healthy controls, were used in the discovery phase. IMRD patients were subdivided into two groups depending on the type of antinuclear antibody (ANA) pattern seen with indirect immunofluorescence: speckled (ANA^{S}) and homogenous (ANA^{H}).
IMRD ANA^{neg}, IMRD patients without antinuclear antibodies
SRMA, steroid-responsive meningitis-arteritis
SLE, systemic lupus erythematosus

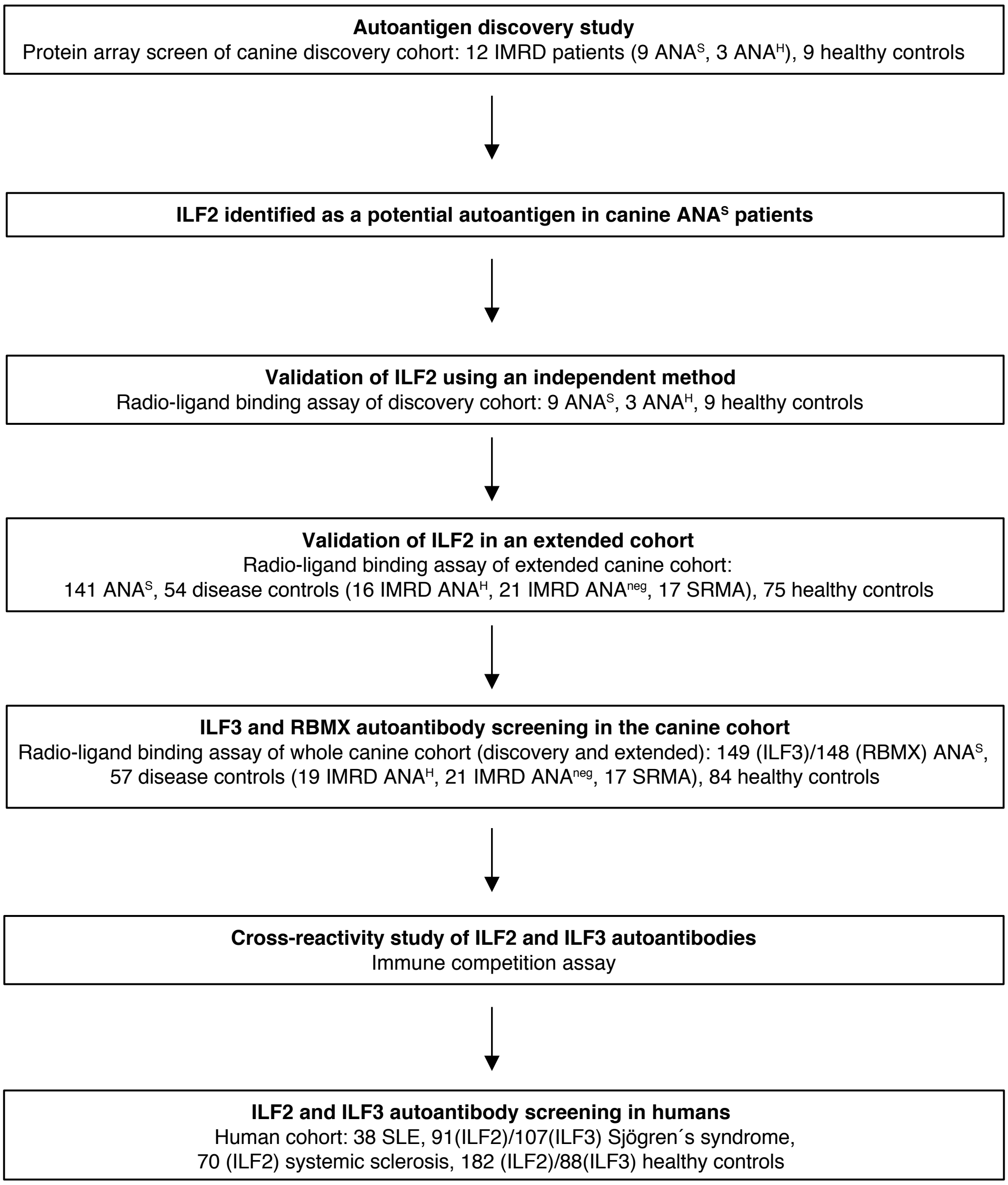

Supplementary Fig. 2. Sample selection in the discovery phase

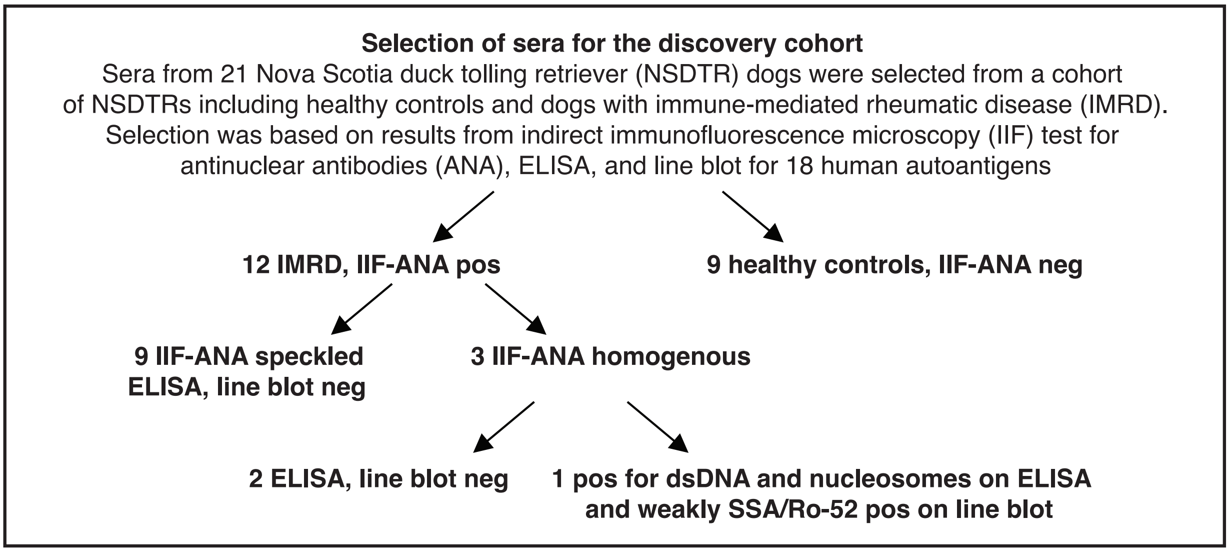

Supplementary Fig. 3. ILF2 and ILF3 autoantibodies do not cross-react
Possible cross-reactivity of ILF2 (a) and ILF3 (b) autoantibodies was investigated by immune competition. Serum from an ILF2- and ILF3-positive patient was used at a dilution of 1:1280. In both experiments, 1 U of labelled protein was added to increasing amounts of unlabelled protein. The y-axis represents the immunoreactivity expressed as percentage of the value measured for 1U labelled protein.
3.a.

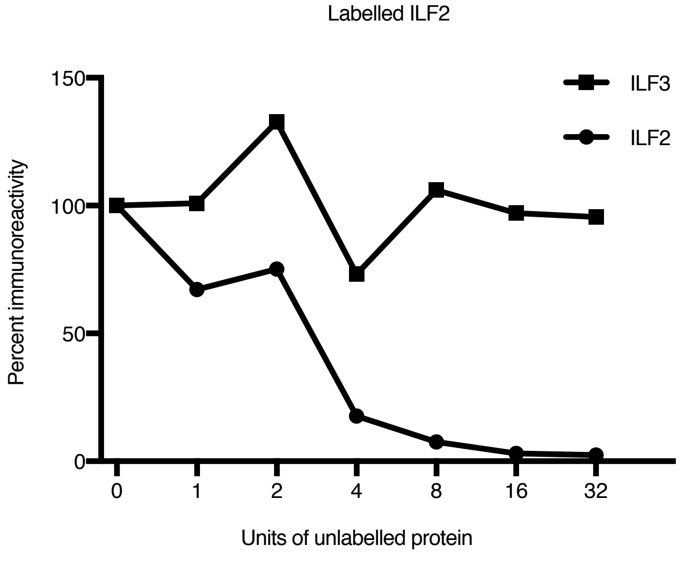

3.b.

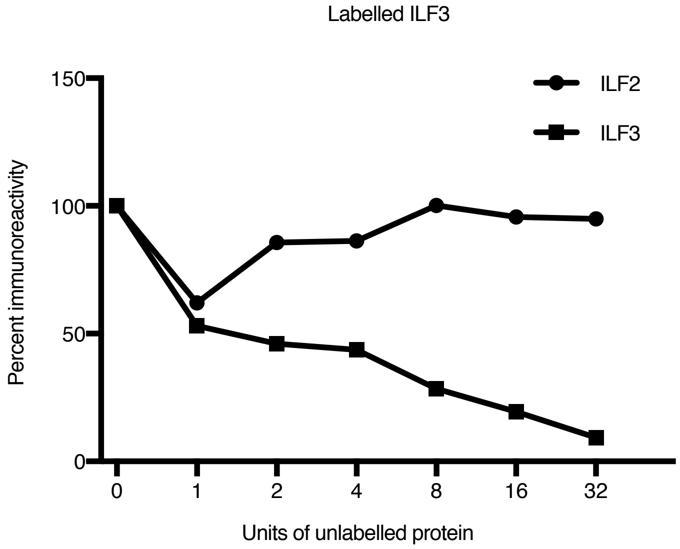

Supplementary Fig. 4. Autoantibodies to ILF2 and ILF3 are sometimes found in human sera
A radio-ligand binding assay was used to screen for ILF2 and ILF3 autoantibodies in sera from human patients with systemic lupus erythematosus (SLE), Sjögren´s syndrome or systemic sclerosis (only ILF2 autoantibodies), and in healthy blood donors. The cutoff value was calculated from healthy controls as the mean + 5 SD. All samples were analysed in duplicate. Autoantibody index = (sample value mean - negative control) / (positive control - negative control) * 100.

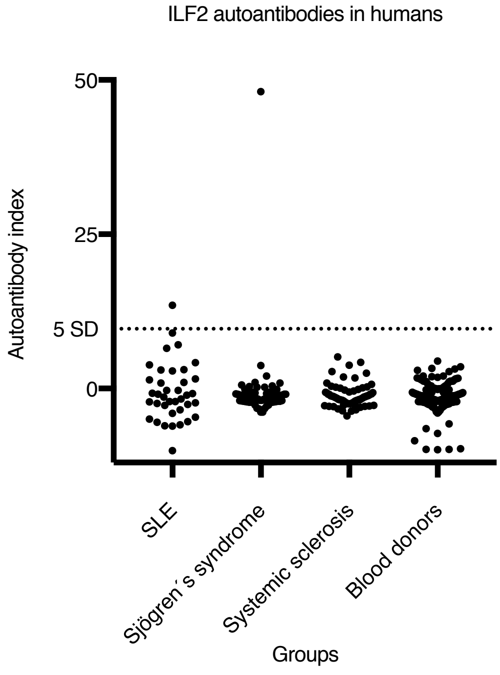

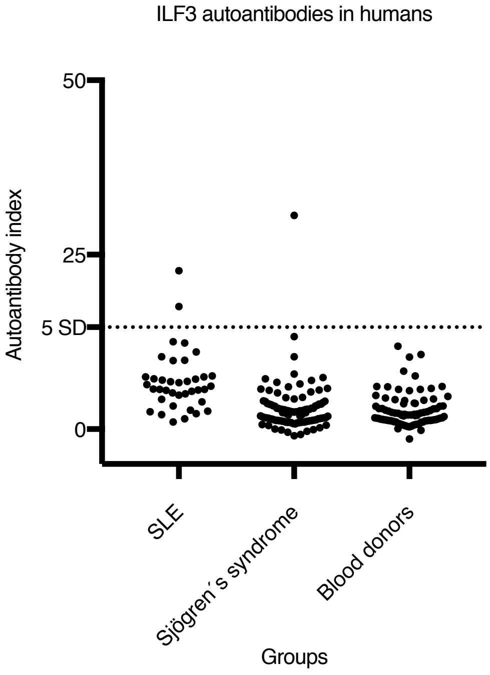

Supplement: Supplementary file 1 — Supplementary information [file 41598_2018_23034_MOESM1_ESM.pdf]
